# Molecular Regulation of Alternative Polyadenylation (APA) within the *Drosophila* Nervous System

**DOI:** 10.1016/j.jmb.2017.03.028

**Published:** 2017-10-27

**Authors:** Raul Vallejos Baier, Joao Picao-Osorio, Claudio R. Alonso

**Affiliations:** Sussex Neuroscience, School of Life Sciences, University of Sussex, Brighton BN1 9QG, UK

**Keywords:** APA, alternative polyadenylation, 3′UTR, 3′ untranslated region, miRNA, microRNA, RBP, RNA-binding protein, CPA, cleavage and polyadenylation, PAS, polyadenylation site, CFI, cleavage factor I, CNS, central nervous system, *Drosophila*, alternative polyadenylation (APA), RNA, nervous system, CNS

## Abstract

Alternative polyadenylation (APA) is a widespread gene regulatory mechanism that generates mRNAs with different 3′-ends, allowing them to interact with different sets of RNA regulators such as microRNAs and RNA-binding proteins. Recent studies have shown that during development, neural tissues produce mRNAs with particularly long 3′UTRs, suggesting that such extensions might be important for neural development and function. Despite this, the mechanisms underlying neural APA are not well understood. Here, we investigate this problem within the *Drosophila* nervous system, focusing on the roles played by general cleavage and polyadenylation factors (CPA factors). In particular, we examine the model that modulations in CPA factor concentration may affect APA during development. For this, we first analyse the expression of the *Drosophila* orthologues of all mammalian CPA factors and note that their expression decreases during embryogenesis. In contrast to this global developmental decrease in CPA factor expression, we see that cleavage factor I (CFI) expression is actually elevated in the late embryonic central nervous system, suggesting that CFI might play a special role in neural tissues. To test this, we use the UAS/Gal4 system to deplete CFI proteins from neural tissue and observe that in this condition, multiple genes switch their APA patterns, demonstrating a role of CFI in APA control during *Drosophila* neural development. Furthermore, analysis of genes with 3′UTR extensions of different length leads us to suggest a novel relation between 3′UTR length and sensitivity to CPA factor expression. Our work thus contributes to the understanding of the mechanisms of APA control within the developing central nervous system.

## Highlights

• We study the expression of the *Drosophila* orthologues of all mammalian CPA factors and note that their expression overall decreases during embryogenesis.
• In contrast, we observe an increase in the expression of the CFI factors CFI25 and CFI68 in the *Drosophila* embryonic nervous system.
• We demonstrate a functional role of CFI expression on APA control during neural development *in vivo*.

## Graphical Abstract

**Figure:**
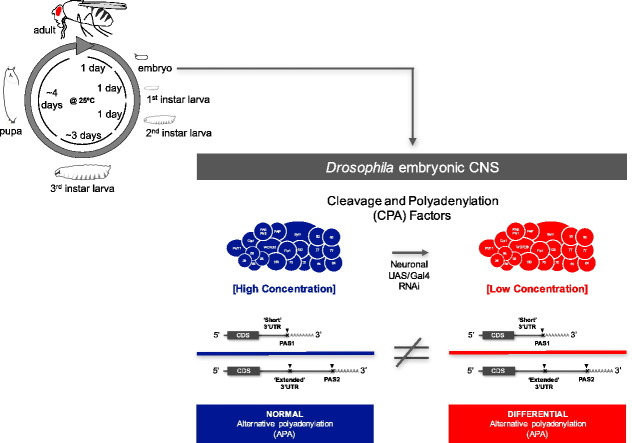

## Introduction

**Fig. 1:**
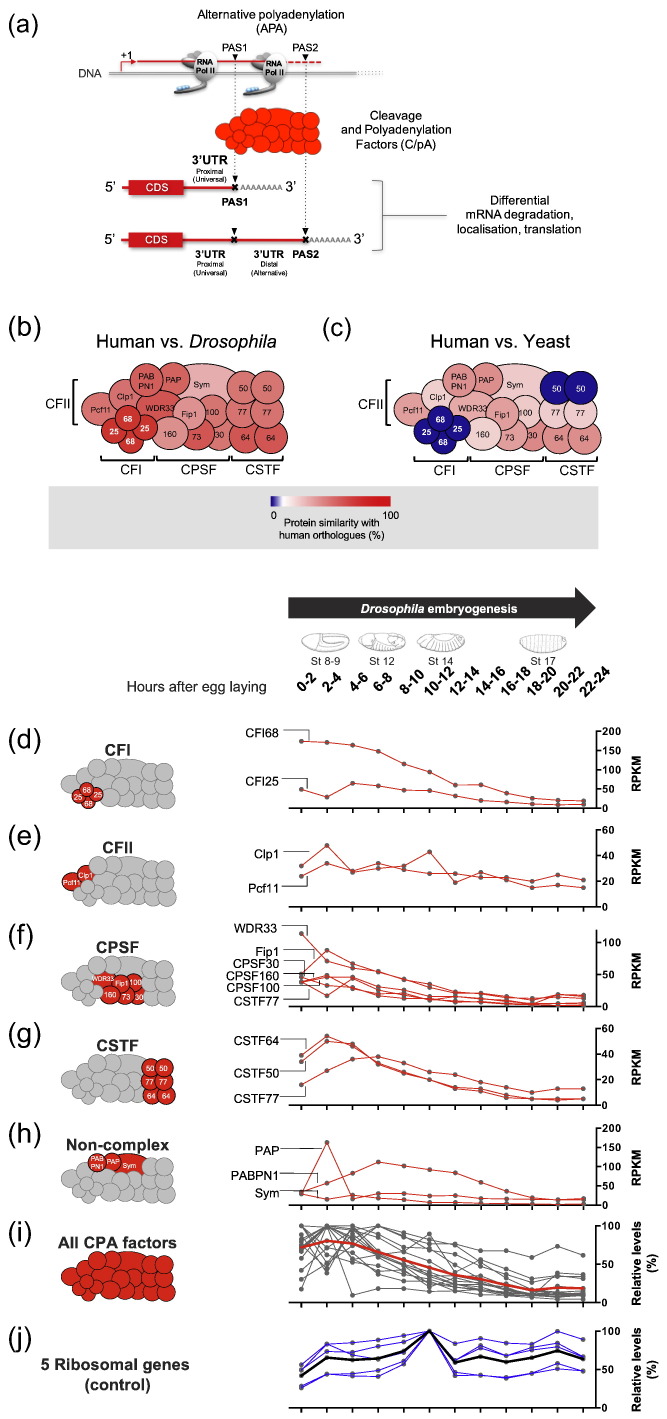
The *Drosophila* CPA machinery is as complex as its human counterpart, and expression of its constituents decreases during embryogenesis. (a) Diagram of the process of APA. During transcription, RNA Pol II goes through different PASs (inverted black triangles), which demark the site for the binding of CPA factors and 3′ end termination, generating mRNAs with different 3′ UTR lengths. (b–c) Diagram of the human CPA machinery showing protein similarity with the orthologues of (a) yeast and (b) *Drosophila* by colour code. Blue colour indicates the absence of orthologues. Notably, all human factors have orthologues in the *Drosophila* genome and they show much higher similarity than their yeast counterparts, with the CFI complex being the most similar between humans and *Drosophila*. Please note that the architecture of the yeast CPA machinery is different and that the features of human complexes were used in all diagrams for simplicity. (d–j) RNA expression levels throughout *Drosophila* embryogenesis measured by Reads Per Kilobase of transcript per Million Mapped Reads (RPKM) values from the modENCODE project [22] (d) of CFI, (e) CFII, (f) CPSF, (g) CSTF, (h) “Non-Complex” factors. All factors together in relative levels with the mean shown in (i) red and (j) five different ribosomal genes as a control in relative levels with the mean shown in blue. Each curve in the diagram represents a member of that complex. On top, embryo diagrams for four representative stages in the indicated time windows (modified from Ref. [50]). Note that the expression levels of the whole CPA machinery show a general trend of high expression levels during early embryogenesis and reduction of expression levels towards late embryogenesis. In contrast, ribosomal genes (blue) do not show this trend and are stably expressed during most of embryogenesis.

Alternative polyadenylation (APA) is an RNA processing mechanism that leads to the generation of mRNA forms bearing different 3′ untranslated regions (3′UTRs; Fig. 1a) [1], [2], [3]. APA is a very prevalent mechanism that affects the majority of genes in a wide variety of metazoans including mammals and insects. Given that 3′UTR sequence composition and structure determine the nature of physical interactions between mRNAs and RNA modulators such as microRNAs (miRNAs) and RNA-binding proteins (RBPs), APA-dependent variations in 3′UTR length and composition are thought to have a significant impact on mRNA dynamics within the cell through effects on mRNA degradation, localisation, and translation (Fig. 1a) [4], [5]. Accurate control of APA patterns may therefore be critical to the organism, particularly within the physiological context of animal development when gene activity must be closely coordinated with complex cell patterning and differentiation processes.

The end point of a given mRNA is determined by two concerted biochemical reactions that involve the endonucleolytic cleavage of the RNA transcript 3′end and the concomitant synthesis of a string of adenosine nucleotides (poly-adenylation) on the free 3′ terminus [1]. These reactions are commonly referred to as the process of cleavage and polyadenylation (CPA), which is controlled by a multiprotein complex composed of CPA factors [6], [7], [8]. The definition of the site for CPA [i.e., the polyadenylation site (PAS)] is achieved through contacts of the CPAs with specific *cis*-elements present in the RNA (Fig. 1a). Among these, the so-called polyadenylation signal (A[A/U]UAAA) [9] emerges as a core element, but many other auxiliary elements are key for proper PAS definition [1]. Furthermore, PAS sequences are redundant, and whether they are used or not also depends on their location within each transcript [10].

APA is believed to emerge—at least in part—as a result of a complex interplay between gene-specific *cis*-elements present in the nascent RNA and their contacts with the CPA machinery and other regulatory factors.

Despite its prevalence and critical roles in gene regulation, the molecular mechanisms underlying APA remain relatively poorly understood, especially *in vivo*. Our work investigates this problem using the fruit fly *Drosophila melanogaster* as a model system. With its powerful genetics and genomics, and the availability of a wide spectrum of tools to manipulate gene function in precise temporal and spatial coordinates, *Drosophila* emerges as an excellent system to study the molecular mechanisms of APA during development.

## Results and Discussion

Previous work in our laboratory [11] and elsewhere [12] has demonstrated that specific *Drosophila* RBPs such as the pan-neural RBP ELAV can affect APA by influencing the kinetics of RNA processing [11], [13] or by affecting transcriptional initiation dynamics [14]. In addition to the roles of specific RBPs, data from mammalian cell cultures [15], [16] suggest that the concentration of general factors involved in cleavage and polyadenylation can play an important role in the definition of APA reactions. Indeed, in previous experiments in *Drosophila*, it was observed that a mutation in the *Drosophila* orthologue of CSTF77 [*Su(F)*] can affect APA in the *Adh-related* transcript [17], as well as APA of its own mRNA [18]. Here, we build on these data and further explore that the model that changes in the expression of general CPA factors can control APA within the physiologically relevant context of *Drosophila* neural development.

As a first step to explore the roles of CPA in APA control in flies, we sought to establish the *Drosophila* orthologues of all the mammalian CPA factors and determine their evolutionary conservation when compared with their human counterparts. Our analysis (Fig. 1b–c) shows that all CPA factors are evolutionarily conserved between humans and fruit flies. This possibly reflects the importance of having a complex CPA machinery for the regulation of the genetic programs underlying both insect and mammalian biology. Despite overall evolutionary conservation, levels of similarity between human and *Drosophila* factors vary from factor to factor. Of special note are the members of the tetrameric cleavage factor I (CFI) complex, CFI25 and CFI68 [19], [20], [21], which are the factors displaying highest conservation between *Drosophila* and human, sharing 77% and 79% similarity at the protein level for CFI25 and CFI68, respectively. This molecular complex binds to pre-mRNAs upstream of the polyadenylation sequence, and its crystal structure suggests that CFI might participate in the selection of different polyadenylation sequences within pre-mRNAs via an RNA-looping mechanism [20]. In contrast to the high level of evolutionary preservation of CFI proteins between flies and humans, the CFI complex is one of the two CPA factors that are absent in yeast (Fig. 1b–c), perhaps reflecting the special roles of these factors in controlling the genetic programs underlying animal development and physiology.

If changes in the concentration of CPA factors play any role within the physiologically relevant context of *Drosophila* development, then we should expect to find variations in the expression level of these proteins during the process of embryogenesis: the transformation of the fertilised egg into a complex organism, bearing multiple tissues and complex cellular patterning. To establish whether this was the case, we analysed RNA sequencing data [22] (modENCODE†) for each one of the *Drosophila* CPA factors during the full period of embryogenesis, which, in *Drosophila*, spans for approximately 22 h if cultures are kept at 25 °C [23]. These observations showed that in contrast to the case of reference genes [i.e., genes encoding ribosomal proteins (Fig. 1j)], the expression of most CPA factors shows a clear decrease as development proceeds, conforming to an apparent global trend to reduce CPA factor expression towards the end of embryogenesis when much of embryonic patterning is largely completed, giving way to cell differentiation processes underlying the formation of complex tissues such as the nervous system (Fig. 1i). A possibility is that this trend might arise from global decay of maternal transcripts; yet, the five ribosomal genes used as a control (Fig. 1j), which are known to be maternally deposited, do not show this trend. This global trend of progressively lower expression levels of CPA factors shows variation between individual complexes. For instance, differences in the expression of CFI68 between early and late embryogenesis are much more pronounced than those observed in the expression of its partner within the CFI complex, CFI25 (Fig. 1d), while the two members of CFII (ClpI and Pcf11) show a similarly slight but nonetheless clear decline in expression levels from early to late time points (Fig. 1e). Yet, despite variations in the embryonic expression patterns of individual CPA factors (Fig. 1d–h), we were unable to detect a single cleavage or polyadenylation factor with increased expression in later stages of embryogenesis. This is in contrast to the profiles of more than 200 genes, including genes with roles in cardiac development and function [e.g., *pericardin* (*prc*), *Sarcolamban A* (*SclA*), *Sarcolamban B* (*SclB*)] [24], [25], as well as genes with roles in the development of the respiratory system [e.g., *waterproof* (*wat*), *windpipe* (*wdp*)] [26], [27], all of which show higher levels of expression at late embryogenesis compared to earlier developmental time points.

**Fig. 2:**
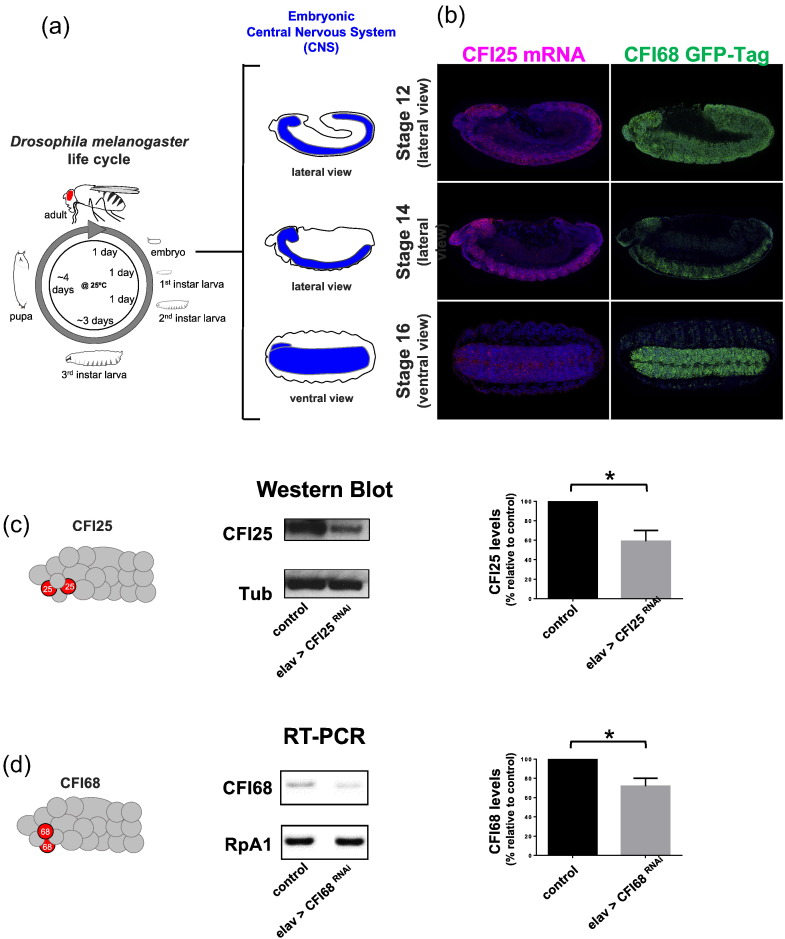
Members of the CFI complex are highly expressed in the nervous system during late embryogenesis. (a, left) Life cycle of *Drosophila melanogaster* at 25 °C (modified from Ref. [50]). Fly embryogenesis takes approximately 1 day to complete and produces a complex organism with multiple organ systems including an elaborate nervous system. (A, right) Schematic representation of the embryonic central nervous system (blue) at stage 12 (top; lateral view), stage 14 (middle; lateral view), and stage 16 (bottom; ventral view). (b) Expression pattern analysis of CFI25 (left, *mRNA* fluorescence in situ hybridisation in magenta) and CFI68 (right, CFI68-GFP reporter stock from Ref. [49] in green) during the embryonic development of the central nervous system. At stage 12 (lateral view), there is ubiquitous expression of both CFI25 and CFI68. However, at stage 14 (lateral view), embryos show a marked enrichment in expression within the central nervous system. During stage 16, the embryos show strong signal in the ventral nerve cord, as shown in a ventral view. We used 4′,6-diamidino-2-phenylindole (DAPI) to label the nuclei. Embryos are oriented with anterior to the left. (c) Diagram representing the location of CFI25 within the CPA machinery; CFI25 levels in stage 16 embryos for the control (elav > +) and the knockdown of CFI25 within the nervous system (elav > CFI25 RNAi), assessed by Western Blot in three independent biological replicates. In each experiment, after normalisation by tubulin, each control was used as 100%, and the value for the knockdown was transformed according to its control in each separate experiment. (d) Diagram representing the location of CFI68 within the CPA machinery; CFI68 levels in stage 16 embryos for the control (elav > +) and the knockdown of CFI68 within the nervous system (elav > CFI68 RNAi), assessed by semi-quantitative RT-PCR in three independent biological replicates. In each experiment, after normalisation by RpA1, each control was also used as 100%, and the value for the knockdown was transformed according to its control in each separate experiment. An unpaired two-tailed *t*-test was used to compare genotypes, * *p* < 0.05.

However, given the complexity at the level of cell and tissue remodelling that takes place during embryogenesis, temporal information is only of limited value when seeking to relate expression profiles to embryonic development. For example, a global decrease in gene expression detected from a combination of all embryonic tissues collapsed in one sample may hide instances where tissues or regions within the organism display constant or even raise levels of gene expression. In this context, the collection of spatial information on expression patterns within the whole intact embryo —for example, by means of gene tagging, immunohistochemistry, or RNA in situ hybridisations— should help in overcoming these limitations and provide much richer information on the dynamic patterns of developmental gene expression and the ways these might relate to the formation and function of specific tissues. With this in mind, we investigated the spatial patterns of CPA expression during *Drosophila* embryogenesis (Fig. 2a), focusing on the analysis of CFI. Our choice of studying CFI in higher detail is based on several considerations: (i) CFI represents one of the hallmarks of metazoan CPA composition, (ii) it affects APA reactions *in vitro* [28], [29], (iii) its molecular structure is well understood in mammals [20], and (iv) one of its members, CFI25, was shown to be involved in human pathologies including cancer and tumorigenesis [30]. Remarkably, expression patterns of both CFI25 and CFI68 showed a clear tissue-specific pattern with higher levels of expression of the two factors within the developing central nervous system (CNS) of the *Drosophila* embryo (Fig. 2b).

**Fig. 3:**
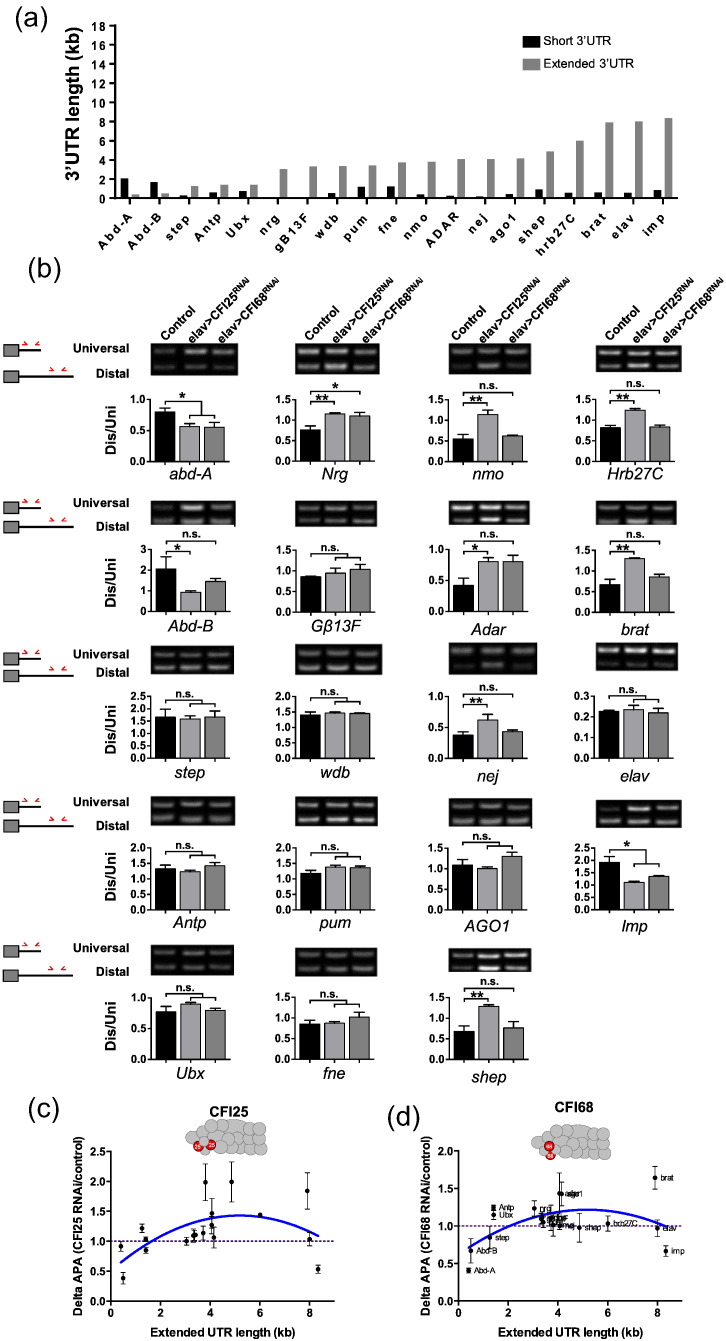
Control of APA by CFI factors during the development of the *Drosophila* embryonic central nervous system. (a) Neural 3′UTR extended genes [31], [32] display a wide spectrum of 3′UTR lengths (grey bars). Length of short 3′UTR isoforms is shown in black. (b) Effects of CFI25 and CFI68 knockdown on APA within the nervous system of *Drosophila* stage 16 embryos. Distal-over-proximal ratios (Dis/Uni) of target genes are shown for wild type and RNAi knockdown of each of the members of CFI within the nervous system (elav > CFI25^RNAi and^ elav > CFI68^RNAi^). Each gene was analysed in three independent biological replicates together with the control in which no RNAi was used (elav > +). For each graph, representative RT-PCR bands of the proximal and distal amplicons are shown for each one of the genotypes. Note that 50% of the genes tested show effects on APA after CFI depletion. Unpaired two-tailed t-tests were used to compare each RNAi against CFI25 or CFI68 with the control; n.s., non-significant. *p* > 0.05; * *p* < 0.05; ** *p* < 0.01. On the left are schematic representations of the short and long 3′UTR isoforms and respective primers (in red) to amplify universal and distal amplicons. Detailed 3′UTR isoforms and primer pairs for each gene are in Fig. S1. (C and D) Correlation between the effects of CFI depletion (*y*-axis, measured as ΔAPA: CFI depletion APA over control APA) and 3′UTR extension [*x*-axis in kilobases (kb)]. Average of ΔAPA values in three biological replicates experiments for the knockdown of (C) CFI25 and (D) CFI68. A dotted line is drawn in the ratio with value 1 to represent the inflexion point in ΔAPA. Error bars represent the SEM. Note that 50% of the genes tested show effects on APA after CFI depletion. Unpaired two-tailed t-tests were used to compare each RNAi against CFI25 or CFI68 with the control; n.s., non-significant. *p* > 0.05; * *p* < 0.05; ** *p* < 0.01. On the left are schematic representations of the short and long 3′UTR isoforms and respective primers (in red) to amplify universal and distal amplicons. Detailed 3′UTR isoforms and primer pairs for each gene are in Fig. S1. (c and d) Correlation between the effects of CFI depletion (*y*-axis, measured as ΔAPA: CFI depletion APA over control APA) and 3′UTR extension [*x*-axis in kilobases (kb)]. Average of ΔAPA values in three biological replicates experiments for the knockdown of (c) CFI25 and (d) CFI68. A dotted line is drawn in the ratio with value 1 to represent the inflexion point in ΔAPA. Error bars represent the Standard Error of the Mean (SEM). Note that genes within the range of 2–6 kb are more susceptible to variations in CFI concentration than genes displaying shorter or longer 3′UTRs. A blue curve represents the trend observed.

Given the particularly high level of expression of CFI factors within the embryonic CNS, we considered the hypothesis that CFI might play a role in APA control, which is known to lead to the production of long 3′UTR mRNA species within neural tissues in both *Drosophila* and mammals [31], [32], [33], [34]. To test this hypothesis, we decided to artificially reduce the expression of CFI25 and CFI68 exclusively within the embryonic CNS. For this, we used the *Drosophila* Gal4/UAS system [35] in combination with RNAi treatments for CFI25 or CFI68 and studied the resulting 3′UTRs produced by a set of genes known to form long 3′UTRs in the CNS. Among these, we included the posterior *Hox* genes, as they provided the very first example of the phenomenon of neural 3′UTR extension in animals [31], together with other neural genes subsequently shown to present 3′UTR extensions [32], [33]. We consciously decided to investigate the effects of CFI expression level on genes expected to produce 3′UTRs of various lengths (Fig. 3a), so that we could examine whether genes forming 3′UTR of different size were equally or differentially sensitive to our treatments *in vivo* (see below). Molecular analysis of the resulting 3′UTRs revealed that more than 50% of the genes tested displayed a change in their APA profiles (Fig. 3b) in conditions shown to significantly reduce the expression of CFI25 and CFI68 (Fig. 2c and d).

Notably, the APA patterns of several genes with known roles in neural differentiation [e.g., *abdominal-A* (*abd-A*), *Abdominal-B* (*Abd-B*), *Neuroglian (Nrg)*, *brain tumour (brat)*] [10], [36], [37], [38] and of genes involved in post-transcriptional regulation [e.g., *Adenosine deaminase acting on RNA (Adar)*] [39] and several RNA-binding proteins [e.g., *alan shepard (shep)*, *IGF-II mRNA-binding protein (Imp)*] [40], [41] were affected in conditions of reduced expression of CFI25 and/or CFI68. Interestingly, some genes showed higher sensitivity to the depletion of one of the CFI factors tested; for example, *Abd-B* APA was strongly affected by CFI25 depletion and much less influenced by CFI68 depletion (Fig. 3b). Yet, other genes were affected by the depletion of either CFI25 or CFI68 [i.e., *abdominal-A* (*abd-A*)*, IGF-II mRNA-binding protein* (*Imp*)*, Neuroglian* (*Nrg*)]. Given that the functional CFI tetrameric complex is composed of two monomers of CFI25 and two monomers of CFI68, the reasons why some genes are sensitive to the depletion of one or both factors are not immediately obvious. Considering that we observe genes that are sensitive to CFI25 depletion only, while seeing no examples of genes exclusively sensitive to CFI68 reduction, one possibility is that expression of CFI25 in the embryonic CNS is more sensitive to RNAi treatment than that of CFI68. Indeed, we do observe that although RNAi treatments for both CFI25 and CFI68 show a statistically significant reduction in expression of the respective factors (Fig. 2c and d), CFI25 RNAi leads to a more pronounced reduction in CFI25 expression than the one observed in CFI68 RNAi conditions. Alternatively, the expression patterns of the genes analysed may only partially overlap with cells expressing CFI complex genes, leaving some or most expression of these genes unaffected by our UAS-RNAi treatments exclusively reducing expression within the CNS by means of the pan-neural *elav-Gal4* driver.

Interestingly, the CFI-dependent changes in APA patterns mentioned above denote, in some cases, an increase in the proportion of distal (alternative) over proximal (constitutive) 3′UTR expression, while in other cases, a decrease in the distal/universal was observed (Fig. 3B). These patterns of APA change by CFI depletion were validated by an independent technique (quantitative RT-PCR; Fig. S2). Although the results of RT-PCR and qPCR are not identical, the great majority of genes (8/10) displayed the same trends in both analyses. The increase in the use of downstream PASs can be interpreted as consistent with the “*first-come, first-served*” model proposed in early IgM 3′UTR processing studies in primary B cell cultures [16], which is based on the notion that proximal PASs are generally weaker than distal PASs [42]. In contrast, shifts from longer to shorter 3′UTR forms observed in other genes in CFI depletion conditions are in line with the results of more recent transcriptome-wide studies in mouse and human cells in culture [15], [43], [44]. A potential explanation for these results is that effects of CFI downregulation are gene-specific, somewhat contributing to the diversification of the mechanisms of gene expression during development.

Taking into consideration that the gene targets analysed displayed 3′UTRs of distinct length, we decided to explore whether 3′UTRs of different length were equally sensitive to CFI depletion. Interestingly, we observed that depletion of both CFI25 and CFI68 (Fig. 3c and d) led to similar trends, suggesting that genes with 3′UTR lengths within the range of 2–6 kb are more susceptible to variations in CFI concentration than genes displaying shorter or longer 3′UTRs. This observation has two implications: (i) that 3′UTRs of distinct length may display different levels of robustness to fluctuations in CPA expression levels, and (ii) that sensitivity to CPA expression levels may be one of the factors that sculpts 3′UTRs during evolution. Although 3′UTR lengths in *Drosophila* show a distribution of sizes with the average around 580 nt, shorter and longer 3′UTRs are expected to reflect—at least to some degree—different biological scenarios. Whilst an increase in 3′UTR sequence up to 1–2 kb can be postulated to expand the plethora of interactions with RNA regulators such as miRNAs and RBPs [4], [31], taking into account the relatively small size of target sequences for miRNAs and RBPs, it seems less likely that 3′UTR extensions in the range of 10–15 kb may play similar roles. Instead, it seems more plausible that such extreme 3′UTR extensions may be linked to the control of mRNA dynamics affecting, for instance, the timing of mRNA release from template DNA or may be involved in RNA localisation events. Despite its modest size, our dataset derived from a subset of neural genes suggests that 3′UTR size may represent a factor that affects sensitivity to CFI expression level. Current experiments in our laboratory involving RNA sequencing analyses of dissected *Drosophila* embryonic and larval CNS samples depleted from CFI and other CPA factors with neural expression should allow us to consolidate these observations across the full transcriptome.

Our work here shows that the expression levels of general CPA factors affect APA patterns of many *Drosophila* neural genes and may therefore play key roles during the unfolding of the genetic programs that underlie the formation of the nervous system. Building on our study and on the high level of evolutionary conservation of fundamental gene regulatory processes across the metazoans, we suggest that variations in CPA levels may also shape neural APA profiles in other organisms, including humans.

## Materials and Methods

### Protein similarity among human, yeast, and *Drosophila* CPA machinery

The sequences of the human proteins were extracted from “Uniprot” [45], the sequences of the yeast proteins were extracted from “Saccharomyces Genome Database” [46], and the sequences of the *Drosophila* proteins were extracted from “Flybase” [47]; when more than one protein isoform was present in each species, we used the most similar ones for the diagram, taking into account the covered region. Protein sequence comparison was done using the NCBI protein‐protein BLAST (BLASTP version 2.5.0) [48].

### CPA factor expression levels throughout embryogenesis

Data were retrieved from the modENCODE project [22] to evaluate the expression levels of all CPA factors during embryogenesis. Gene expression levels are measured as “Reads Per Kilobase of transcript per Million Mapped Reads” and plotted against 2-h time windows from 0 to 24 h, covering the full period of embryogenesis.

### *Drosophila* stocks

Fruit flies (*D. melanogaster*) were cultured using molasses food following standard procedures at 25 °C on a 12-h light and dark cycle. The following stocks were used: CFI68 GFP reporter (y^1^ w*; CG7185[38575]::2XTY1-SGFP-V5-preTEV-BLRP-3XFLAG; Vienna stock center #318105; [49]), RNAi against CFI25 (y* w^1118^; UAS–CG3689 RNAi - Vienna stock center #105499/KK), RNAi against CFI68 (y^1^ sc^⁎^ v^1^; P{TRiP.HMS00113}attP2 - Bloomington stock center #34804), elav-Gal4 driver (P{GAL4-elav.L}2/CyO - Bloomington stock center #8765 and *wild type* stock (Oregon Red).

### Fluorescence in situ hybridisation and immunocytochemistry

Embryos were fixed in 4% formaldehyde using standard protocols. RNA fluorescence in situ hybridisation for CFI25 mRNA was performed as previously described [11]. Briefly, templates of RNA probes were obtained from PCR-amplified embryonic cDNA with the primers 5′-CGTCCAGCCGGTTAATTT-3′ and 5′- GTTAGGTAGCGCTATCGTTG-3′ (probe length of 955 bp) and cloned into pGEM-T vector (Promega). RNA probes were labelled with digoxigenin using the RNA Labelling Kit (Roche) according to the manufacturer's instructions. Fluorescent detection of RNA probes was done using anti-digoxigenin-POD (1:500, Roche) followed by Cy3 TSA amplification kit (1:50, Perkin Elmer). Antibody immunostainings were performed following standard protocols using the primary antibody rabbit anti-GFP (Life Technologies; 1:500) and secondary antibody anti-rabbit-A488 (Life Technologies; 1:750). All embryos were counterstained with 4′,6-diamidino-2-phenylindole (DAPI) to label nuclei and mounted in Vectashield (Vector Laboratories). Fluorescent imaging was carried out using Leica SP8 confocal microscope. All images were processed and analysed in ImageJ.

### Western blotting

We homogenised 20–50 embryos in 20 μl of 2X Laemmli buffer [4% SDS, 20% glycerol, 0.004% bromophenol blue, 0.125 M Tris–HCl (pH 6.8)]; 2 μl of β-mercaptoethanol was added to the samples and boiled at 95 °C for 5 min. Proteins were separated on a 12% SDS-PAGE gel at 100 V for 2 h and then electrophoretically transferred onto a 0.45-μm nitrocellulose membrane (GE Healthcare Life Sciences). After protein transfer, membranes were blocked in 5% milk PBST (1XPBS 0.1% Tween 20) for 1 h and then incubated with primary antibodies overnight at 4 °C. Membranes were washed and incubated with 1:3000 anti-mouse-HRP or anti-rabbit HRP secondary antibodies in 5% milk PBST for 1 h. Detection was performed using Clarity Western ECL Substrate (Bio-Rad) according to manufacturer's instructions. Primary antibodies used were rabbit anti-CFI25 (1:1000, Abcam) and mouse anti-tubulin (1:500, Developmental studies hybridoma bank). Secondary antibodies used were anti-mouse-HRP (1:3000, Cell Signalling Technology) and anti-rabbit-HRP (1:3000, Dako).

### Semi-quantitative RT-PCR

Total RNA was extracted from staged embryos using TRI Reagent (Sigma) followed by RNase-free DNase I treatment (New England Biolabs). We used 1 μg of total RNA for cDNA synthesis oligo(dT) primers (Invitrogen) and MuLV Reverse Transcriptase (Invitrogen) according to the manufacturer's instructions. The same amount of RNA was used when comparing different genotypes. PCRs were performed with primers described in Table S1. Expression values were normalised using reference gene RpA1. At least three independent biological replicates were done for each experiment. All experiments included two negative controls: (i) PCR with 1 μl of No-RT reaction to control for genomic DNA contamination from each RT reaction, and (ii) PCR with nuclease-free water to control for PCR mix contamination. All experiments also included one positive control: Genomic DNA template to control for PCR reaction and primer binding.

### RT-qPCR

We used 200 ng of total RNA for cDNA synthesis with the same protocol used for previous RT-PCR experiments. PCR reactions were done using SYBR green mix 2X (Roche) to a final volume of 20 μl using three biological replicates and two technical replicates per reaction. PCRs were performed using an Applied Biosystems StepOne Plus qPCR machine. Expression values were normalised using reference gene Rp49. All primers were tested for efficiency by using fivefold serial dilutions of template cDNAs. Quantitative PCRs were performed with primers described in Table S2. All experiments included two negative controls: (i) PCR with 1 μl of No-RT reaction to control for genomic DNA contamination from each RT reaction, and (ii) PCR with nuclease-free water to control for PCR mix contamination. All experiments also included one positive control: Control 1:5 cDNA dilution template to control for PCR reaction and amplification curve.

The following are the supplementary data related to this article:

Fig. S1 Diagrams of genes used in this study representing 3′UTR lengths and location of primers. (A–S) Simplified gene structure diagrams for (A) *abd-A*, (B) *Nrg*, (C) *nmo*, (D) *Hrb27C*, (E) *Abd-B*, (F) *Gβ13F*, (G) *Adar*, (H) *brat*, (I) *step*, (J) *wdb*, (K) *nej*, (L) *elav*, (M) *AntP*, (N) *pum*, (O) *AGO1*, (P) *Imp*, (Q) *Ubx*, (R) *fne***,** and (S) *shep* representing short and long 3′UTR isoforms. Primers targeting universal 3′UTRs are shown as blue arrows, and primers for distal 3′UTRs are shown as red arrows. Fig. S1.

Fig. S2 RT-qPCR validation of semi-quantitative RT-PCR results. (A) Diagram showing the genes tested in this study and the genes with affected APA patterns (ΔAPA) after RNAi treatment against CFI25 or CFI68 assessed by semi-quantitative RT-PCR experiments. (B) No RT controls for *RpA1* for cDNAs used in this study to assess APA in neural extended genes by semi-quantitative RT-PCR. The same reaction with the positive RT samples is shown for reference in an agarose gel stained with ethidium bromide. (C) No RT controls for *Rp49* for cDNAs used in this study to assess APA in neural extended genes by RT-qPCR. The same reaction with the positive RT samples is shown for reference in an amplification curve. (D–M) Normalised Dis/Uni values obtained by semi-quantitative RT-PCR (black bars) and by RT-qPCR (grey bars) showed in arbitrary units (a.u.) for (D) *abd-A*, (E) *Abd-B*, (F) *nrg*, (G) *Adar*, (H) *nej*, (I) *Hrb27C*, (J) *brat*, (K) *Imp*, (L) *nmo*, and (M) *shep*. All experiments were done using biological triplicates; error bars represent the standard error of the mean (SEM). Fig. S2.

Supplementary tables Image 2

## Footnotes

† http://www.modencode.org
