# Supplementary material for: Molecular Regulation of Alternative Polyadenylation (APA) within the Drosophila Nervous System
Source: J Mol Biol. 2017 Oct 27;429(21):3290–300. doi: 10.1016/j.jmb.2017.03.028 (PMC5656104; doi:10.1016/j.jmb.2017.03.028)
Supplement: Fig. S1 — Diagrams of genes used in this study representing 3′UTR lengths and location of primers. (A–S) Simplified gene structure diagrams for (A) abd-A, (B) Nrg, (C) nmo, (D) Hrb27C, (E) Abd-B, (F) Gβ13F, (G) Adar, (H) brat, (I) step, (J) wdb, (K) nej, (L) elav, (M) AntP, (N) pum, (O) AGO1, (P) Imp, (Q) Ubx, (R) fne, and (S) shep representing short and long 3′UTR isoforms. Primers targeting universal 3′UTRs are shown as blue arrows, and primers for distal 3′UTRs are shown as red arrows. [file mmc1.pdf]

Figure S1
Vallejos Baier *et al.*

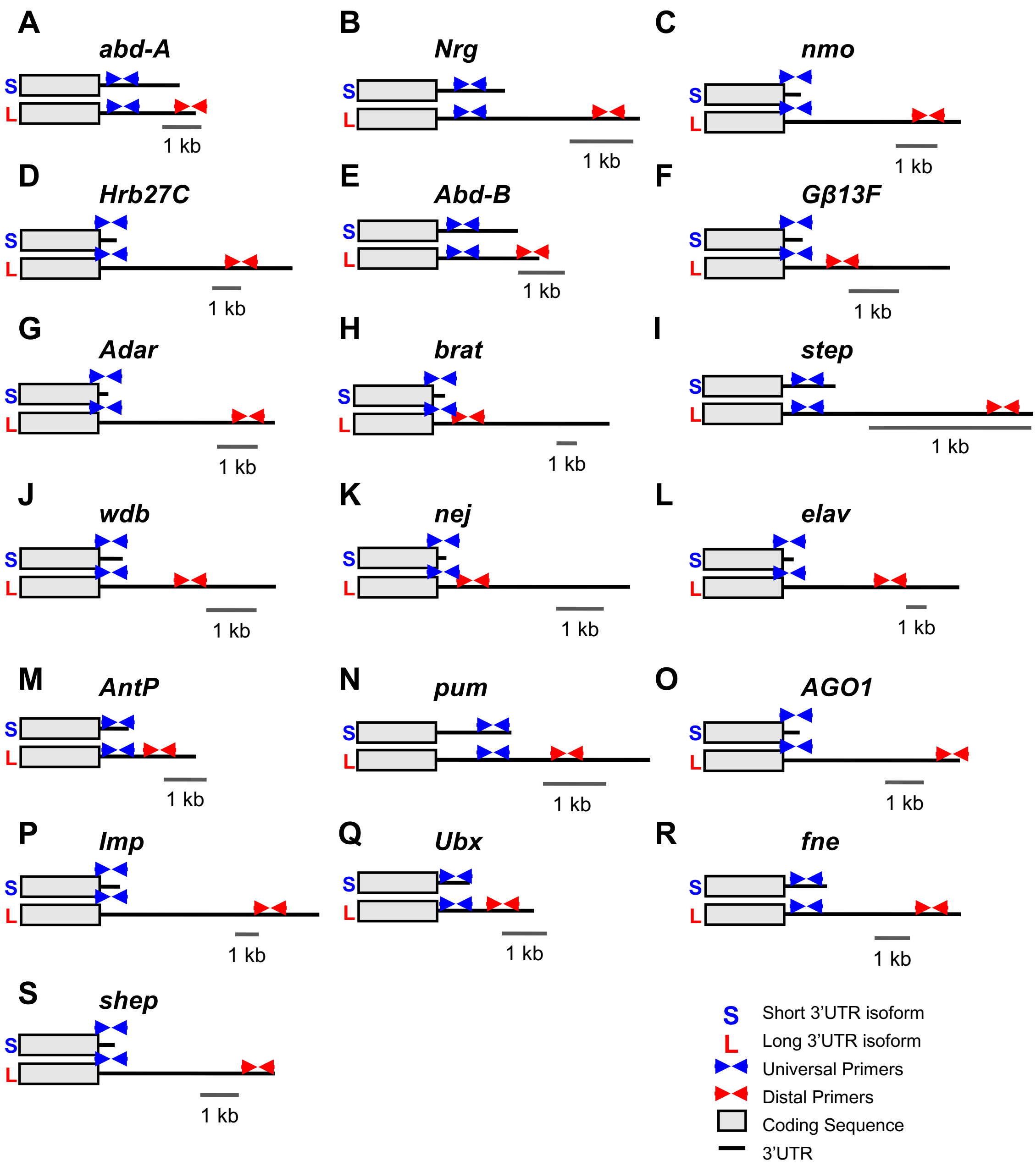

Figure S1. Diagrams of genes used in this study representing 3'UTR lengths and location of primers. (A-S) Simplified gene structure diagrams for *abd-A* **(A)**, *Nrg* **(B)**, *nmo* **(C)**, *Hrb27C* **(D)**, *Abd-B* **(E)**, *Gβ13F* **(F)**, *Adar* **(G)**, *brat* **(H)**, *step* **(I)**, *wdb* **(J)**, *nej* **(K)**, *elav* **(L)**, *AntP* **(M)**, *pum* **(N)**, *AGO1* **(O)**, *Imp* **(P)**, *Ubx* **(Q)**, *fne* **(R)** and *shep* **(S)** representing short and long 3'UTR isoforms. Primers targeting universal 3'UTRs are shown as blue arrows and primers for distal 3'UTRs are shown as red arrows.
