# Supplementary material for: Molecular Regulation of Alternative Polyadenylation (APA) within the Drosophila Nervous System
Source: J Mol Biol. 2017 Oct 27;429(21):3290–300. doi: 10.1016/j.jmb.2017.03.028 (PMC5656104; doi:10.1016/j.jmb.2017.03.028)
Supplement: Fig. S2 — RT-qPCR validation of semi-quantitative RT-PCR results. (A) Diagram showing the genes tested in this study and the genes with affected APA patterns (ΔAPA) after RNAi treatment against CFI25 or CFI68 assessed by semi-quantitative RT-PCR experiments. (B) No RT controls for RpA1 for cDNAs used in this study to assess APA in neural extended genes by semi-quantitative RT-PCR. The same reaction with the positive RT samples is shown for reference in an agarose gel stained with ethidium bromide. (C) No RT controls for Rp49 for cDNAs used in this study to assess APA in neural extended genes by RT-qPCR. The same reaction with the positive RT samples is shown for reference in an amplification curve. (D–M) Normalised Dis/Uni values obtained by semi-quantitative RT-PCR (black bars) and by RT-qPCR (grey bars) showed in arbitrary units (a.u.) for (D) abd-A, (E) Abd-B, (F) nrg, (G) Adar, (H) nej, (I) Hrb27C, (J) brat, (K) Imp, (L) nmo, and (M) shep. All experiments were done using biological triplicates; error bars represent the standard error of the mean (SEM). [file mmc2.pdf]

Figure S2
Vallejos Baier *et al.*

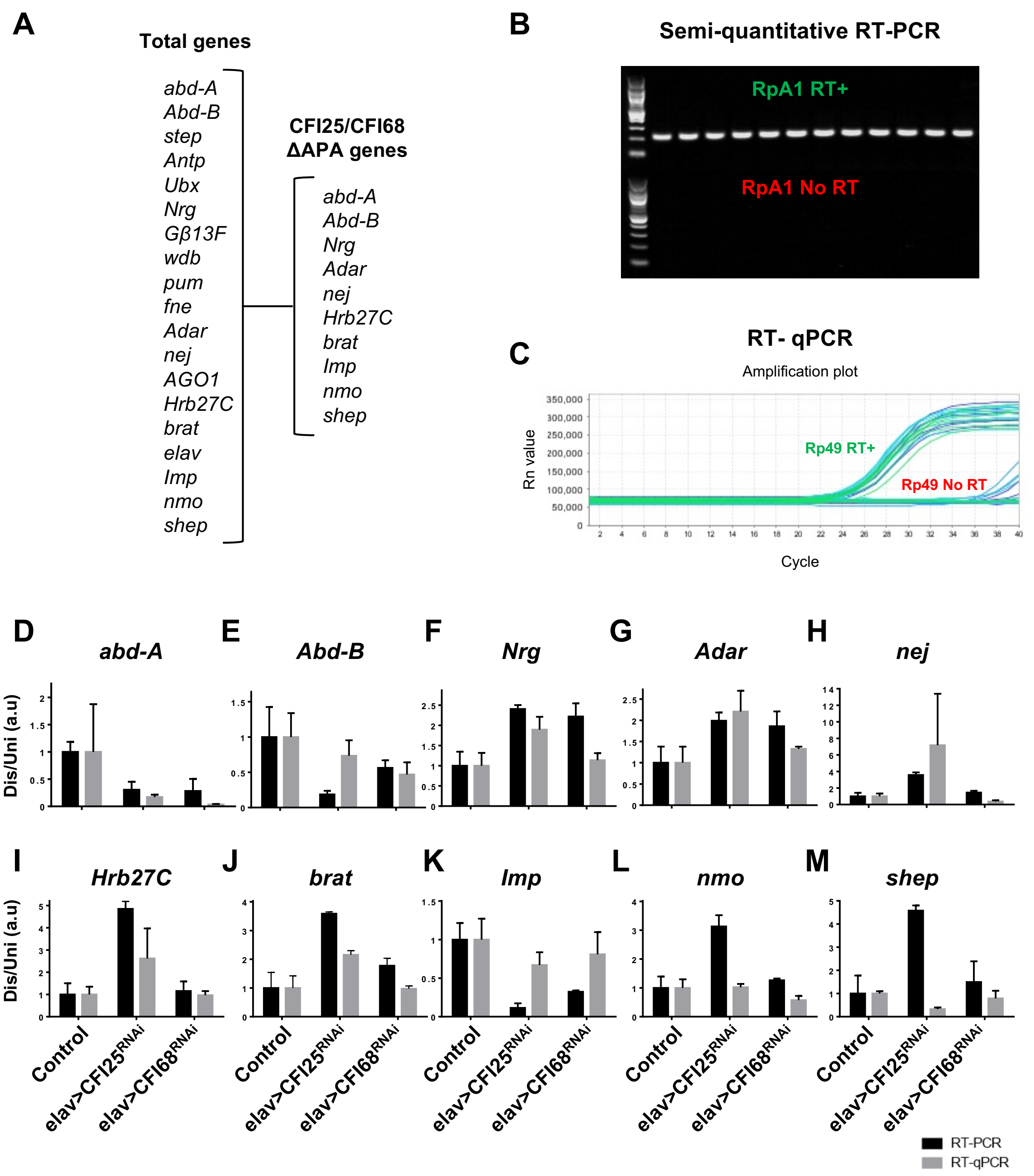

Figure S2. RT-qPCR validation of semi-quantitative RT-PCR results. (A) Diagram showing the genes tested in this study and the genes with affected APA patterns (ΔAPA) after RNAi treatment against CFI25 or CFI68 assessed by semi-quantitative RT-PCR experiments. **(B)** No RT controls for *RpA1* for cDNAs used in this study to assess APA in neural extended genes by semi-quantitative RT-PCR. The same reaction with the positive RT samples is shown for reference in an agarose gel stained with ethidium bromide. **(C)** No RT controls for *Rp49* for cDNAs used in this study to assess APA in neural extended genes by RT-qPCR. The same reaction with the positive RT samples is shown for reference in an amplification curve. **(D-M)** Normalized Dis/Uni values obtained by semi-quantitative RT-PCR (black bars) and by RT-qPCR (grey bars) showed in arbitrary units (a.u.) for *abd-A* **(D)**, *Abd-B* **(E)**, *nrg* **(F)**, *Adar* **(G)**, *nej* **(H)**, *Hrb27C* **(I)**, *brat* **(J)**, *Imp* **(K)**, *nmo* **(L)** and *shep* **(M)**. All experiments were done using biological triplicates, error bars represent the SEM.
