# Supplementary material for: Molecular Regulation of Alternative Polyadenylation (APA) within the Drosophila Nervous System
Source: J Mol Biol. 2017 Oct 27;429(21):3290–300. doi: 10.1016/j.jmb.2017.03.028 (PMC5656104; doi:10.1016/j.jmb.2017.03.028)
Supplement: Supplementary file 3 — Supplementary tables [file mmc3.docx]

| **Table S1: Semi-quantitative RT-PCR primers** | | | | |
| --- | --- | --- | --- | --- |
| **Gene** | **Forward primer (5’ to 3’)** | **Reverse primer (5’ to 3’)** | **Amplicon length (bp)** | **Source** |
| Ubx Universal | GAAATGACGCGGAGACAGAT | AATCTGCGCTCCTTCCACTA | 236 | [31] |
| Ubx Distal | GAACGAAGGCAGATGCAAAT | GGTAAGTGGTCGGATGCAGT | 225 | [31] |
| AbdA Universal | CGGGTTTTATTGCTGTGGAT | CGTTGGCCCAGAGACTCTAC | 193 | [31] |
| AbdA Distal | CCTTTTCGATGAGGTCCAAA | CGGTTTCGGTCGGTCTAATA | 219 | [31] |
| Antp Universal | ACGGAGTCTACCCACTTAAA | GATCTGAGGTCACATGAGTTG | 336 | This study |
| Antp Distal | GAGGACGGAATGGCAAACTA | GTCTTTTCACCTGGGATTGG | 165 | [31] |
| AbdB Universal | CGTATTTCTCTCAACGCTCTC | CGGAGTGTGTCTTCTTGTTT | 300 | This study |
| AbdB Distal | TCCGTACAACACCATTTTCG | AGTGGCGATTACGAGCTGAT | 229 | [31] |
| Elav Universal | AGTAGCAGGCAGGAGAAA | GACTGTGCCAACCTTTGA | 303 | This study |
| Elav Distal | GACGAACTGCTCCGATTT | CGCTCTTCTCCGATTACTTAC | 284 | This study |
| ADAR Universal | TGTATATGCTAAGTTCAGTTTACG | GCTTAAAGTGCTTGTTTATAATGTG | 223 | This study |
| ADAR Distal | CCCGCTAAACCAGTGATAAG | GCGTTTAGCCCAGAATGT | 319 | This study |
| Nrg Universal | GAACAACAAGCAACACAACA | GAGCGGGACAAAGATATACAG | 320 | This study |
| Nrg Distal | CGAATCGGTTCGGCTTTAT | GAGGCTGGGTATTGGTTATTC | 300 | This study |
| Pum Universal | GCATACACCCACACAATGA | TTGGCTTACTTGGCTAACAG | 318 | This study |
| Pum Distal | GCAGGGCTCGGTATTATTT | TTCGCTGGCTTACACTAAC | 330 | This study |
| Imp Universal | AGCACCCACCACAATTTAC | GCGCGCTGCTTTCTATTA | 320 | This study |
| Imp Distal | GGAACGAAACGAAACGAAAC | GCTCAGTCTCCAGTTGATTAC | 282 | This study |
| Ago1 Universal | CCACTTCCTTCCCTCAAATC | CAAACTTGTGCCTGACATTC | 281 | This study |
| Ago1 Distal | ATGCGAGTTTGTGAAATATGC | GGGTACATTTCGTGGGTTTA | 244 | This study |
| Brat Universal | CGGTCTCTCCAGCTCTAAT | CGAGGGTTTGAAGTGAGAAG | 341 | This study |
| Brat Distal | CTTGAGGATGTGTGTGCATAG | CCGTGTGGCTTTCGTATTT | 294 | This study |
| Wdb Universal | CCAAGATCAGTAAGAGCGTAAG | ATTAACGCGGACACACAC | 305 | This study |
| Wdb Distal | CTAAGCGACGTGTGTGTAAG | CAAACAGGTCGAGTCGATAAG | 307 | This study |
| Nmo Universal | AGAACATGGAGGAGAGGAG | TACCGCTGCTGCTTTAAC | 298 | This study |
| Nmo Distal | AAACACTCGATACGCTAACC | CTTTGTTGCGTGCCTTTAC | 290 | This study |
| Fne Universal | AGATGAGCCAGACGACAA | GAGTTATGCTGGTAGTTCCTAAA | 296 | This study |
| Fne Distal | GCCCAGCAGCTAATGAAA | GGGTGTGTAAGTGTGAACTG | 302 | This study |
| Nej Universal | CAGCTACAATGGTTGGTAGG | GTTGGTCTTCGTCGTCATC | 103 | This study |
| Nej Distal | CATAGGGATCGGGATTAGGA | GCGTCGTTGTTGTGTTTG | 311 | This study |
| Gβ13F Universal | GAAACAGAAACAGCAGCATAAG | GTTGTTGTGGTCTACGTTCTA | 304 | This study |
| Gβ13F Distal | GGCCAGTCAGTCAGTTAATC | GGTTTCCTCCATCTTCATCTT | 303 | This study |
| Shep Universal | ACCCAGCATCCAGAATCTA | CTCACTTGCCGCTGTTT | 299 | This study |
| Shep Distal | ACCCACACCAAATAGTTTCC | GCGTTCATTCCTCCTCTATG | 306 | This study |
| Step Universal | CAGCTCGGCGAATCTTT | TGATGGCTTGTTTCGAGTC | 227 | This study |
| Step Distal | CGGCTTACTGACGTCTAATC | TTACCGCCGTCCTTTATATTC | 311 | This study |
| Hrb27C Universal | CAGCACTCTCACCCATTTAG | TGGTATTTCGCGCTCTATTC | 286 | This study |
| Hrb27C Distal | GATGCGCCAAATGCAAAC | ACTCTCGTTCTGAGGGATTAG | 317 | This study |
| RpA1 | AAGAGCATCGACGACCTGAT | GCCACATTCAACCGCTTATT | 213 | This study |

| Table S2: RT-qPCR primers | | | | |
| --- | --- | --- | --- | --- |
| Gene | **Forward primer (5’ to 3’)** | **Reverse primer (5’ to 3’)** | **Amplicon length (bp)** | **Source** |
| ADAR Universal | AAATGTACGCTTTACCAGCA | TCGTATACGGTTAATGAGCACAAA | 75 | This study |
| ADAR Distal | TTTGTCCGAATCGTCTTAATCTCA | ATATCGCAAGAAGTCGGTACTCAA | 67 | [32] |
| Brat  Universal | TGGTCCGGCACTCACTACTTC | TGGAGAGACCGAGCAAGTCA | 64 | [32] |
| Brat  Distal | TGGGCAACAATCAGGATATGG | GCGCAACAGATCAAGAGGATATG | 62 | [32] |
| Imp  Universal | CCACAGCACACCCATAAGATTC | TGTAAATTGTGGTGGGTGCTTT | 60 | [32] |
| Imp  Distal | GGTTTTCCTCTCTCCTTTCCAAA | CGAAACGATCGGGATTATAGGAT | 63 | [32] |
| Nej  Universal | GAGGGCTAGGGCTAGACTGAATG | CGGTTGGTCTTCGTCGTCAT | 74 | [32] |
| Nej  Distal | CCCCGCCCCATGTGT | AATTAGGAACGCGCACAGACA | 73 | [32] |
| Nmo  Universal | CCTTCGCCTCACCAATGG | TTTTGATTTTAGGCAGCAATCG | 59 | [32] |
| Nmo  Distal | CATCTTCGACTCCACAGTGAATG | CAAAGCAGCCAGCTTCTGTCT | 68 | [32] |
| Nrg  Universal | TATCGAAACCGAGCCCTTA | TCTGCTGTGATGGTGTTTG | 66 | This study |
| Nrg  Distal | GCTTCAGCATAGTTTTCAATCGAAT | TGTGGGCTCGGTTTTTGG | 61 | [32] |
| Hrb27C  Universal | CTCGGCGAATGATGAAGAAG | CGAACAGACGACGCTTTAG | 65 | This study |
| Hrb27C Distal | GCGGTACACTTCTGATAACG | CTCTCGTTCTGAGGGATTAGA | 100 | This study |
| Shep  Universal | AAAGCCATCCCGTAATGC | CACATCCAACCGCCTTATT | 78 | This study |
| Shep Distal | CGACGAGGAGTGTTCTAATC | GTTCCCAGTTTGGTTTCTTG | 85 | This study |
| Abd-A  Universal | CGGGTTTTATTGCTGTGGAT | CGTTGGCCCAGAGACTCTAC | 193 | [31] |
| Abd-A  Distal | CCTTTTCGATGAGGTCCAAA | CGGTTTCGGTCGGTCTAATA | 219 | [31] |
| Abd-B  Universal | GCTAGTCCAGCGATTGGAAG | GTCGGTTGGTCACACATCAG | 177 | [31] |
| Abd-B  Distal | TCCGTACAACACCATTTTCG | AGTGGCGATTACGAGCTGAT | 229 | [31] |
| Rp49 | CCAGTCGGATCGATATGCTAA | TCTGCATGAGCAGGACCTC | 268 | [31] |
